# Congenital Diaphragmatic Hernia With Poor Clinical Outcome: Key Lessons To Be Learned

**DOI:** 10.7759/cureus.71628

**Published:** 2024-10-16

**Authors:** Shoaib Shahzad Khan, Hadia Aslam, Malik Shahbaz, Gul-e-Rana Abdul Manna, Aqsa Khan, Atif A Hashmi

**Affiliations:** 1 Pediatrics, Canadian Specialist Hospital, Dubai, ARE; 2 Internal Medicine, Dr. Hassan's Hospital, Abuja, NGA; 3 Pathology, Liaquat National Hospital and Medical College, Karachi, PAK

**Keywords:** congenital anomalies, congenital diaphragmatic hernia, diaphragmatic hernias, prenatal diagnosis, pulmonary hypoplasia

## Abstract

Congenital diaphragmatic hernia (CDH) refers to the abnormal protrusion of abdominal contents (stomach, intestine) into the thoracic cavity, leading to the underdevelopment of the lungs (pulmonary hypoplasia). It is a critical neonatal condition that presents significant challenges in both diagnosis and management, especially in resource-limited countries.

This case report describes a term female baby born via normal vaginal delivery to a mother with no prenatal care. The baby showed no respiratory effort and required resuscitation. Despite intubation, the baby’s air entry remained poor, and she was transferred to the NICU. Initial blood gas analysis revealed severe respiratory and metabolic acidosis (pH: 6.8, pCO2: 86), indicating significant respiratory compromise. A chest X-ray confirmed the diagnosis of right-sided CDH, accompanied by left-sided pneumothorax, hypoplastic lungs, and a compressed heart. The patient was stabilized in the NICU with high-frequency ventilation and was subsequently transferred to a referral center with a Level IV NICU, where she was kept on high intermittent positive pressure ventilation (IPPV). Ultimately, the neonate did not survive the postoperative period, succumbing to the severe complications associated with her condition.

This case report discusses the presentation, management, and outcomes of a female neonate born with CDH, requiring immediate intervention. Despite aggressive resuscitation efforts and surgical repair, the neonate succumbed to severe complications. This case underscores the importance of early detection, prompt treatment, and the complexities involved in managing CDH, particularly in resource-limited settings.

## Introduction

Congenital diaphragmatic hernia (CDH) is a life-threatening condition in which abdominal contents herniate into the thoracic cavity due to impaired diaphragmatic development, leading to insufficient lung development. CDH occurs in approximately 0.7 to 15.9 per 10,000 births [1]. Statistics have shown that CDH is an economic burden, with significant costs in both developed and developing countries [2]. It typically presents with cyanosis (blue discoloration of the body), respiratory distress, displaced heart sounds, and a scaphoid abdomen. The condition is characterized by small, underdeveloped lungs (pulmonary hypoplasia) and persistent pulmonary hypertension, a failure of the normal transition of circulation that occurs after birth, leading to marked pulmonary hypertension and hypoxemia. This complicates postnatal management and increases the risk of mortality. In addition to affecting lung development, CDH can also lead to left ventricular hypoplasia [3] and is often associated with vascular remodeling (intimal and medial thickening of proximal pulmonary arteries leading to vaso-occlusive lesions) which causes pulmonary hypertension. In some cases, CDH can be linked with other congenital defects such as congenital heart defects [4]. The precise epidemiology and etiology of CDH are not fully understood, though genetic factors are believed to play a role [5,6]. The condition is associated with high mortality, and survivors might suffer from long-term morbidity, gastroesophageal reflux, persistent pulmonary hypertension, and neurodevelopmental impairment [7]. Therefore, it is imperative to diagnose this condition early through good prenatal care and frequent fetal imaging.

## Case presentation

Initial presentation

The patient, a female neonate born at term via normal vaginal delivery to a mother with no prenatal care, presented with respiratory failure at birth requiring immediate resuscitation. Despite intubation and mechanical ventilation, poor air entry necessitated transfer to the NICU.

Diagnostic workup

**Figure 1:**
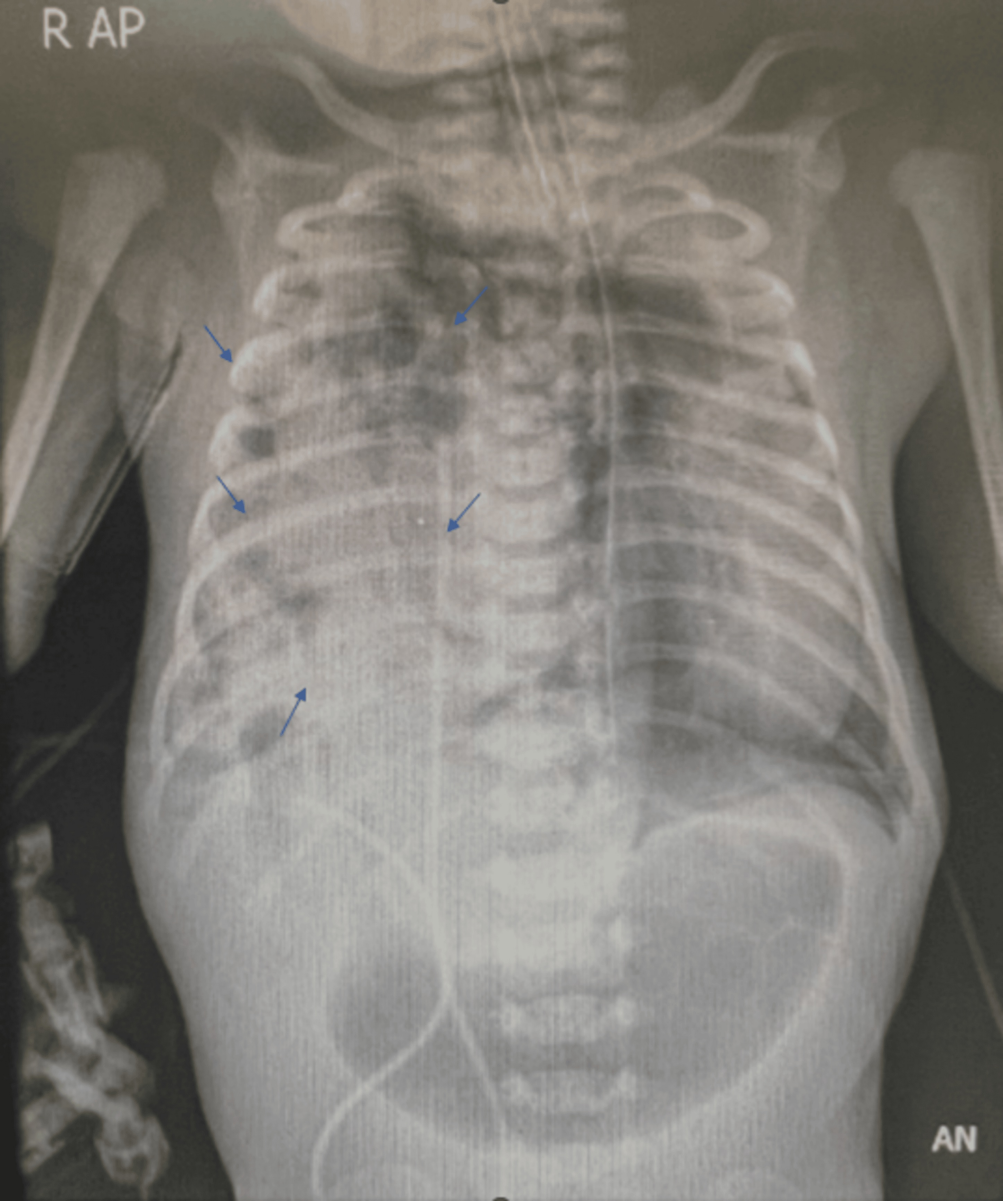
Chest X-ray AP view. Chest X-ray AP view showing the right hemithorax filled with multiple radiolucent regions continuous with the abdominal cavity (arrows), with interval displacement of the mediastinal structures and cardiac silhouette to the opposite side (left). No convincing gas-containing loops of bowel can be seen in the abdomen. These findings implicate multiple loops of bowel occupying the right hemithorax due to the absence of the right hemidiaphragm, resulting from congenital diaphragmatic hernia. AP: Antero-posterior.

Initial arterial blood gas analysis showed severe metabolic and respiratory acidosis (pH: 6.8, pCO2: 86), indicating significant respiratory compromise. A chest X-ray confirmed right-sided CDH, accompanied by left-sided pneumothorax, hypoplastic lungs, and a compressed heart (Figure 1).

A 2D echocardiogram performed within 24 hours post-birth revealed the heart displaced to the left, with patent ductus arteriosus (PDA) and a persistent foramen ovale (PFO), causing a right-to-left shunt. Findings also indicated severe persistent pulmonary hypertension of the newborn (PPHN), including a dilated right atrium and ventricle, paradoxical ventricular septum movement, and right-to-left shunting via the PFO and PDA.

Management

The patient was stabilized in the NICU with high-frequency jet ventilation and was subsequently transferred to a referral center with a Level IV NICU, where she was kept on high intermittent positive pressure ventilation (IPPV). These steps were taken by the clinicians as the patient was in respiratory failure and was thought be the standard management at that time. Despite these efforts, her condition remained critical. Since the hospital was not equipped with extracorporeal membrane oxygenation, it necessitated transfer to another child care hospital for further stabilization with nitric oxide therapy and high IPPV. At 96 hours of age, she underwent surgical repair of diaphragmatic defect. The decision of surgical repair was taken as there was no significant improvement on medical management. Procedure, which lasted seven hours, was complicated by severe hypothermia, acidosis, and hypotension.

Outcome

Despite aggressive management including nitric oxide therapy, high IPPV, and surgical intervention, the neonate succumbed to severe complications associated with CDH in the postoperative period.

Our case did not have pre-natal diagnosis of CDH, however absence of respiratory effort at birth prompted some investigations (X-ray and ECHO) that confirmed the diagnosis. This case underscores the critical importance of prenatal care and imaging, early recognition of symptoms (e.g., absence of respiratory effort, displaced heart sounds), and the severe complications associated with CDH that necessitate rapid and comprehensive management.

## Discussion

Pathophysiology and diagnosis

CDH arises due to a failure of the diaphragm to close during embryonic development, typically around the sixth week of gestation, which is the first stage of lung development [8]. This defect allows abdominal organs, including the intestines, stomach, and liver, to herniate into the thoracic cavity, leading to compromised lung development and pulmonary hypoplasia [9]. Approximately 85% of cases are left-sided (Bochdalek hernia), while right-sided hernias, which often involve the liver and large bowel, occur in about 13% of cases, as observed in this case [10].

Variable degrees of pulmonary hypoplasia linked to a reduced pulmonary vasculature cross-sectional area and an altered surfactant system are characteristic features of CDH. Dysfunction in the surfactant system further decreases the already small alveolar capillary membrane necessary for gas exchange. The severity of CDH is directly related to the degree of pulmonary hypoplasia and associated anomalies, such as PPHN, as seen in this patient [11].

Prenatal CDH is most frequently diagnosed during the 20-week prenatal ultrasound, although it can be detected as early as 16 weeks. Postnatally, infants commonly exhibit a scaphoid abdomen, respiratory distress (symptoms may include cyanosis, retractions, grunting respiration), and a barrel-shaped chest. Lung auscultation may show poor air entry on the left in cases of left-sided postero-lateral hernia, while cardiac sounds may shift to the right side of the chest. Features of pneumothorax, such as poor perfusion and poor air entry, might be observed in severe defects. High frequencies of anomalies associated with CDH occur. Genetic syndromes like Marfan syndrome, Donnai-Barrow syndrome, Pallister-Killian mosaic syndrome, and Fryns syndrome are associated with CDH, while dysmorphisms like spinal dysraphism, and extremities or craniofacial abnormalities might indicate a syndromic form of CDH [12].

Management and treatment

The management of CDH requires a multidisciplinary approach aimed at stabilizing the neonate's respiratory and hemodynamic status [13]. Immediate intubation and avoidance of mask ventilation are crucial to prevent further air entry into the gastrointestinal tract, which can exacerbate lung compression [14]. High-frequency ventilation is employed to manage PPHN and optimize oxygenation [15]. The management strategy encompasses various facets, including fetal interventions, delivery room management, ventilation strategies, pH management, hemodynamic stabilization, timing of surgical repair, anesthetic considerations, and long-term outcomes [16].

In this case, the neonate underwent multiple transfers to specialized centers for advanced respiratory support and surgical intervention. The timing of surgical repair is controversial; some experts advocate for early repair within 24 hours of stabilization, while others recommend delaying surgery until pulmonary artery pressures have normalized [17]. Despite timely interventions, severe complications such as hypothermia and acidosis developed, contributing to the poor outcome.

In CDH, medical therapy is aimed to optimize oxygenation whilst avoid barotrauma. The management of CDH includes placing a vented orogastric tube connected to continuous suction to prevent bowel distention and additional lung compression, immediate tracheal intubation to avoid mask ventilation, synchronized ventilation with the baby’s respiratory effort to avoid high peak inspiratory pressures, continuous monitoring of blood pressure, perfusion, and oxygenation, and maintaining ionized calcium and glucose levels within the reference range [13].

Surgical options in the postnatal period include closing the diaphragmatic defect and reducing herniated viscera, using chest tube drainage in cases of tension pneumothorax, and, rarely, single lung transplantation. The ideal timing for CDH repair varies, with some studies suggesting repair as early as 24 hours after stabilization, while others find a 7-10 day delay to be generally well-tolerated. Some surgeons prefer to operate as soon as pulmonary arterial pressure is stable for about 24 to 48 hours based on echocardiographic findings [17].

Pharmacotherapy for CDH aims to stabilize circulating volume and blood pressure, alleviate pulmonary distress, and correct hypoxemia. This includes the administration of opioid analgesics (e.g., fentanyl), vasoactive agents (e.g., dobutamine, dopamine, milrinone), pulmonary vasodilating agents (e.g., nitric oxide), neuromuscular blocking agents (e.g., vecuronium, pancuronium), and other pH management strategies such as optimal FiO2, epinephrine, consideration for milrinone, PGE1 infusion, and other pH-specific medications like sildenafil [13].

Fetal surgery in severe CDH

Fetal surgery for severe CDH can be a preemptive treatment that allows the fetal lungs to develop sufficiently before birth, enhancing the newborn's chance of survival and subsequent quality of life. Fetoscopic Endoluminal Tracheal Occlusion (FETO) is a procedure aimed at improving outcomes for fetuses with severe CDH [18]. The indications for FETO include isolated left CDH (L-CDH), severe pulmonary hypoplasia with an observed/expected lung-to-head ratio (O/E LHR) of less than 25%, gestational age under 30 weeks (with the procedure typically performed between 27 and 29 weeks), a normal fetal karyotype, and maternal age over 18 years.

FETO is considered to potentially increase the survival rates of fetuses with CDH. Data supporting its efficacy were sparse until the conduct of large randomized controlled trials, such as the TOTAL trials. These studies showed that the survival rates for fetuses with moderate to severe CDH were higher in the FETO group compared to the control group [19].

Extracorporeal membrane oxygenation (ECMO)

In cases where post-delivery neonates present with fragile or severely compromised lungs, ECMO may be required. ECMO is employed as a last resort when conventional treatment options are inadequate. This technology performs the work of the lungs, allowing them to rest and heal, thereby facilitating the surgical repair of CDH. However, the use of ECMO is associated with serious risks, including infection and bleeding, which underscores the need for careful management by experienced specialists [20].

Postnatal surgery for CDH

Postnatal surgery for CDH can occur as early as three days of life, depending on the baby's condition after birth. Babies with CDH are extremely sensitive to noise and movement. During the procedure, babies receive general anesthesia and continuous monitoring by a pediatric anesthesiologist. An incision is made just below the baby's rib cage, the organs in the chest are repositioned into the abdomen, and the hole in the diaphragm is sewn closed. This creates space in the chest, allowing the lungs to continue growing; children continue to develop more alveoli into early childhood. For babies with large defects or those completely lacking a diaphragm, the hole is closed with a GORE-TEX® patch or a muscle flap. Sometimes the abdominal wall cannot be closed during surgery. In these cases, temporary placement of a silo, mesh, or Vacuum Assisted Closure® (VAC) device may be recommended. As the child grows, the condition of the patch is regularly monitored by doctors to ensure that it remains intact [8].

Prognosis and long-term outcomes

The prognosis for neonates with CDH is influenced by several factors, including the size and position of the diaphragmatic defect, the presence of liver herniation, and the degree of pulmonary hypoplasia [21]. The overall survival rate for CDH ranges from 40% to 62%, with higher mortality rates observed in cases involving right-sided hernias and severe PPHN [22]. Long-term outcomes for survivors include chronic lung disease, neurodevelopmental delays, and gastrointestinal complications, necessitating ongoing multidisciplinary follow-up [23].

This case illustrates the significant challenges associated with managing CDH, particularly in cases involving severe pulmonary hypoplasia and PPHN. Despite advances in neonatal care, the mortality rate for CDH remains high, underscoring the need for continued research into early detection, fetal intervention, and optimized postnatal management strategies [24]. Our case had a poor outcome owing to delayed access to optimal care and frequent hospital transfers. Tables 1-2 compare the treatment and outcomes of our case with previous literature.

**Table 1:** Comparison of outcome/survival in this case report with previous literature. CDH: Congenital Diaphragmatic Hernia; PPHN: Persistent Pulmonary Hypertension of the Newborn; ECMO: Extracorporeal Membrane Oxygenation; VIS: Vasoactive-Inotropic Score.

Study/Case Report	Patient Characteristics	Surgical Intervention	Key Complications	Outcome	Survival Rate (%)
This Case Report (2024)	Female neonate, term, right-sided CDH, left pneumothorax	Surgical repair at 96 hours of age	Pulmonary hypoplasia, PPHN, severe acidosis	Neonate did not survive post-surgery	Expired
Paoletti et al. (2020) [1]	Neonates with CDH, varying hernia sizes	Surgical repair within first 7-10 days	Persistent pulmonary hypertension	40%-62% survival depending on severity	40%-62%
Politis et al. (2021) [24]	Multi-country study, children with CDH	Varying surgical timing	High PPHN and hypoxia	Mortality in right-sided hernias, especially severe cases	38%-50%
Glenn et al. (2019) [20]	Neonates with CDH on ECMO	Early surgical repair on ECMO	Severe complications of ECMO (infection, bleeding)	Improved survival with early ECMO repair	70%
Liu et al. (2023) [21]	Neonates with severe CDH	Surgery within 24 hours post-birth	High vasoactive-inotropic score (VIS)	Higher VIS correlated with poor prognosis	60%

**Table 2:** Comparison of outcome of this case report with findings from other research studies on CDH. CDH: Congenital Diaphragmatic Hernia; PPHN: Persistent Pulmonary Hypertension of the Newborn; ECMO: Extracorporeal Membrane Oxygenation; VIS: Vasoactive-Inotropic Score.

Research Study	Focus of Study	Key Interventions	Key Findings	Outcome/Survival Rate (%)
This Case Report (2024)	Term neonate with right-sided CDH	Surgical repair, high-frequency ventilation	Neonate succumbed post-surgery due to complications	Expired
Wong et al. (2018) [3]	Prognostic markers and long-term outcomes in CDH patients	Surgical intervention, nitric oxide therapy	Pulmonary hypertension is a key factor in prognosis; delayed surgery has mixed results	55%-60%
Dao et al. (2021) [17]	ECMO and early CDH repair	Surgical repair after ECMO	Early repair improved survival but complications were common	65%-70%
Mandell et al. (2021) [14]	Persistent pulmonary hypertension in newborns with CDH	Use of vasoactive agents, nitric oxide, ECMO	Vasoactive-inotropic score (VIS) helped predict mortality	60%-65%
Chatterjee et al. (2020) [16]	Management and treatment outcomes of CDH	Timing of surgery, ECMO, nitric oxide therapy	Delayed repair until pulmonary pressure normalization improved	62%

Our case emphasizes the need for prenatal care in resource-limited countries. Early prenatal diagnosis can facilitate treatment planning, prevent diagnostic delays, and improve outcomes. Additionally, we stress the importance of recognizing signs and symptoms at birth, such as lack of respiratory effort, and utilizing simple X-ray imaging and echocardiographic parameters to diagnose this condition promptly.

## Conclusions

CDH remains a challenging condition with a high risk of mortality, particularly in cases involving severe pulmonary hypoplasia and right-sided hernias. This case report highlights the importance of early diagnosis, prompt intervention, and the complexities of managing CDH in neonates. Despite aggressive treatment efforts, the outcome for this patient was unfavorable, reflecting the critical nature of the condition and the need for ongoing advancements in neonatal care and surgical techniques. Comparison with previous literature underscores the importance of early diagnosis and prompt management. The adverse outcome in our case, compared with previous studies, was mainly due to the lack of prenatal diagnosis and delayed management. Future research should focus on improving prenatal diagnostic methods, refining surgical timing, and developing new therapeutic approaches to enhance survival rates and long-term outcomes for affected infants.
